# The Climates and Baths of Great Britain

**Published:** 1896-12

**Authors:** 


**The Climates and Baths of Great Britain.** Vol. I. Pp. xvi., 640. London: Macmillan and Co. 1895.

This work, being the report of a Committee of the Royal Medical and Chirurgical Society of London, comes to us with authority. The Committee was appointed in 1889, and is now carrying on its investigations under the Chairmanship of Dr. W. M. Ord, with Dr. A. E. Garrod as Honorary Secretary. The volume deals with the climatology of the south coast of England and the balneology of the more important spas of Great Britain. Much information was obtained from medical men practising in the various localities, and much also by the personal investigation of various members of the Committee. As each county is made the subject of an independent essay of some twenty to fifty pages in length, which contains full details of temperature, moisture, sunshine, mortality, &c., there must of necessity be much useless repetition, and the book, as one to read, is more tiresome than the dictionary of "the late lamented Johnson"; but as a book for consultation on the details in the climatology of any particular district it will satisfy the cravings of the most exacting enquirer, and will give him even more information than he can accept without a degree of reservation. When we read that "acute renal dropsy is reported to be practically unknown in Bournemouth," we cannot avoid the inference that such cases may have been classified under the heading of Influenza or some similar convenient appellation. The differences in the climatology of adjoining counties must be so small that it seems to have been necessary to magnify them in a work like this.

In the chapters on the Medicinal Springs of Great Britain we find no mention of the Hotwells of Clifton, nor is Clifton mentioned as a place which has a climate of any kind; but there is time to remedy this omission in the next volume. The neighbouring city of Bath "claims our first attention by reason of its antique renown, and by reason of the high temperature and abundant flow of its springs . . . For the complete and thorough application of a hot water, with no pronounced chemical constituents, Bath has at the present time an armament which, so far as our experience goes, is not excelled, if, indeed, it be equalled, by what exists in any similar resort in Great Britain or elsewhere." It is added: "No one can doubt that gout, anæmia, neuralgia, and many diseases of the skin can here find powerful methods of relief." Dr. W. M. Ord and Dr. A. E. Garrod have given us a very complete and interesting account of Bath and its thermal waters, with a judicial summary of their therapeutic uses.

